# Biomarkers of Central Nervous System Involvement from Epithelial Ovarian Cancer

**DOI:** 10.3390/cells10123408

**Published:** 2021-12-03

**Authors:** Giulia Scotto, Fulvio Borella, Margherita Turinetto, Valentina Tuninetti, Anna A. Valsecchi, Gaia Giannone, Stefano Cosma, Chiara Benedetto, Giorgio Valabrega

**Affiliations:** 1 Department of Oncology, University of Torino, 10123 Torino, Italy; giulia.scotto@ircc.it (G.S.); margherita.turinetto@ircc.it (M.T.); valentina.tuninetti@ircc.it (V.T.); annaamela.valsecchi@ircc.it (A.A.V.); gaia.giannone@ircc.it (G.G.); 2 Division of Medical Oncology, Candiolo Cancer Institute, FPO-IRCCS, 10060 Candiolo, Italy; 3 Gynecology and Obstetrics 1, Department of Surgical Sciences, City of Health and Science, University of Turin, 10126 Turin, Italy; fulvio.borella87@gmail.com (F.B.); stefano.cosma@unito.it (S.C.); chiara.benedetto@unito.it (C.B.)

**Keywords:** brain metastases, ovarian cancer, biomarker

## Abstract

Epithelial ovarian cancer (EOC) is the leading cause of death among women affected by gynaecological malignancies. Most patients show advanced disease at diagnosis (FIGO stage III-IV) and, despite the introduction of new therapeutic options, most women experience relapses. In most cases, recurrence is abdominal-pelvic; however, EOC can occasionally metastasize to distant organs, including the central nervous system. The incidence of brain metastases (BMs) from EOC is low, but it has grown over time; currently, there are no follow-up strategies available. In the last decade, a few biomarkers able to predict the risk of developing BMs from OC or as potential therapeutic targets have been investigated by several authors; to date, none have entered clinical practice. The purpose of this review is to offer a summary on the role of the most relevant predictors of central nervous system (CNS) involvement (hormone receptors; BRCA; MRD1; PD-1/PD-L1) and to highlight possible therapeutic strategies for the management of metastatic brain disease in EOC

## 1. Introduction

Brain metastases (BMs) are the most frequent type of intracranial cancer in adults [1] and more than 100,000 patients with BMs are diagnosed every year in the U.S. [2].

Lung cancer, breast cancer, melanoma, and renal cell carcinoma are the tumors that most frequently spread to the central nervous system [3].

BMs diagnoses have been increasing in recent years [4,5]; this could be explained by improvements in neuroimaging technology, a general increase in cancer incidence and, for many cancer types, an improvement in prognosis, with a longer overall survival rate, which may lead to a higher incidence of BMs over the natural history of the disease [6,7].

Epithelial ovarian cancer (EOC) is the first cause of death among women with gynaecological malignancies in the U.S. in 2021 [8].

In recent years, it has become increasingly clear that serous tubal intraepithelial carcinoma (STIC) is the immediate precursor of high grade serous ovarian cancer (HGSOC) [9]. This new paradigm of ovarian cancer genesis was based on the observation of dysplastic epithelium in the fallopian tubes in women with breast cancer gene 1 (BRCA1) and BRCA2 germline mutations [10].

The development of a tumor protein p53 (TP53) mutation, the earliest known molecular alteration of HGSOC, leads to the development, within decades, of a STIC, followed, in a few years, by progression to an HGSOC. Genetic predisposition (such as deficit of homologous recombination) accelerates the process.

EOC often remains localized in the abdomen and pelvis, with lymphatic diffusion being very common: at diagnosis, approximately 70% of patients present pelvic and/or paraortic lymph nodes involvement [11,12]. On the contrary, haematological spread is a late event; the most common distant sites of metastasis are pleura (33%), liver (26%), and lung (15%) [13]. Locations like heart, bones, and skin tend to be rarer.

The central nervous system (CNS) is involved in 0.3–12% of different series [11,14,15,16,17,18,19] and prognosis of patients with ovarian cancer disseminated to the brain is poor, with a median overall survival of 10.1 months [18].

Favourable prognostic factors in patients with BMs from EOC include: younger age, a good Karnofsky performance status at time of diagnosis, serous histology, absence of extracranial lesions and multimodal treatments, which include surgery, radiotherapy and systemic chemotherapy [16,17,18,20,21,22]. The chemotherapeutic agents used are the same utilized for the treatment of ovarian cancer, in particular the platinum salts ± docetaxel, due to their ability to pass the blood–brain barrier (BBB). Less information is known about single chemotherapic agents, such as gemcitabine and trabectedin [16,17,18].

Unfortunately, no validated biomarker is currently able to predict CNS spread from EOC.

The aim of this review is to present a comprehensive and updated overview of molecular, pathological characterization and known biomarkers of BMs derived from ovarian cancer.

## 2. Histopathological Features

Serous EOC is the histotype most frequently associated with CNS metastases [23]. Histological features of BMs from EOC usually mimic the primary tumor [18]: they mainly show a predominant papillary pattern with psammoma bodies; the glands are slit-like, with prominent cellular budding and bizarre nuclei [24]. The immunophenotypic pattern of HGSOC is well known: Cytokeratin 7 (CK7) and low molecular weight keratin (LMWK) are present in almost 100% of cases; nuclear expression of paired box protein 8 (PAX8) is present in 80%; Cytokeratin 20 (CK20) may be focally expressed in a few cases [25,26]. The estrogen receptor (ER) is frequently present, while the progesterone receptor (PgR) is often weakly positive or negative [27,28]. Strong expression of Wilms’ tumor 1 (WT1), p53 and p16 are typical of HGSOC [29,30].

Nafisi et al. proposed a multi-step immunohistochemical approach, in order to discriminate between serous, endometrioid and clear cell histology of metastatic ovarian carcinoma to the CNS [23].

The first step consists in establishing Mullerian origin, with the immunohistochemical positivity of CK7, PAX8 and ER (except clear cell carcinoma) and the lack of CK20. The strong, diffused positivity of WT1 orients towards the HGSOC (step 2), and the nuclear intense staining of p53 and p16 confirms the diagnosis (step3). If WT1 is not expressed, the lack of ER and the presence of Hepatocyte nuclear factor-1β (HNF-1β) staining more likely configure a clear cell histology (step 4), while an ER focal nuclear staining directs towards endometrioid ovarian cancer (step 5) [23].

## 3. Biomarkers of Brain Metastases from Epithelial Ovarian Cancer

Table 1 summarizes the currently known biomarkers of brain metastases from EOC.

## 4. Hormone Receptors

The possibly predictive role of hormone receptors in EOC spreading to the SNC has been analysed.

Estrogens produce biological effects by interacting with two main estrogen receptors (ERs) isoforms: ERα and ERβ.

**Figure 1:**
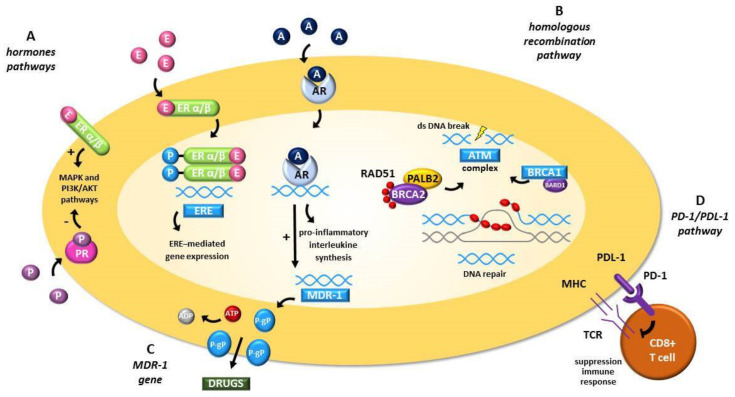
Pathways and proteins identified in brain metastasis from ovarian cancer. (**A**) Hormones pathway. Effects of estrogens are mediated by activation of nuclear receptors, the estrogen receptor-α (ER-α), and estrogen receptor-β (ER-β). The complex estrogen and ERα/β, phosphorylated and dimerized, moves into the nucleus and activates transcription of a battery of genes (ERE-genes), which stimulate cell proliferation. However, complex estrogen-ER, through transmembrane protein, activates the MAPK and PI3K/AKT pathway. Indeed the association progesterone and PR, regulates these pathways. Finally, AR causes the synthesis of pro-inflammatory interleukins (IL-6 and IL-8) and activates the ABC transporters expression, as MDR-1. (**B**) *Homologous recombination pathway*. The complex ATM recognizes dsDNA break and recruits complex BRCA1 and BRCA2, blocking the replication fork. RAD51 forms a nucleoprotein filament that mediates homologous pairing to the sister chromatid to repair the dsDNA break. (**C**) *MDR-1 gene*. P-glycoprotein (P-gp), the product of the MDR1 gene, is an ATP-binding cassette (ABC) efflux transporter. This pump can efflux cytotoxic agents using ATP driven energy. (**D**) *PD-1/PD-L1 pathway*. The interaction between PD-1 on CD8 positive T cell and its ligands PD-L1 on tumor cell inhibits TCR signalling, leading to reduced T cell proliferation and cytokine production and increasing susceptibility to apoptosis. Legend. E: estrogen; ER: estrogen receptor; ERE: estrogen responsive element; P: progesterone; PR: progesterone receptor; A: androgen; AR: androgen receptor; IL: interleukin; MDR: Multi drug resistance; P-gp: P-glycoprotein; TCR: T cell receptor; ABC: ATP-binding cassette; MHC: major histocompatibility complex.

ERα is overexpressed in metastatic ovarian cancer tissue [36] and its activation induces cell proliferation and enhances the epithelial-mesenchymal transition (EMT) process by increasing the migratory capacity of cells, facilitating the formation of distant metastases [37]. Additionally, ERα can trigger mitogen-activated protein kinase (MAPK) and fosfatidilinositolo 3-kinasi-Akt (PI3K-Akt) pathways leading to cell proliferation and neoplastic transformation [38] (see Figure 1).

On the other hand, ERβ expression in OC appears to have antitumoral effects [39], via the increase of the number of cells in G2/M phase, and by enhancing apoptosis, interfering with ERα activity [40,41,42].

Furthermore, loss of ERβ expression is linked with an increased risk for developing distant metastasis [42].

The progesterone receptor (PgR) also exerts an antineoplastic effect on cell migration and invasion by regulating the PIK3/AKT pathway (Figure 1), inhibiting EMT and leading to apoptosis through down-regulation of B-cell lymphoma 2 (bcl-2) and upregulation of the mRNA level of p53 [38].

Finally, the androgen receptor (AR) can promote OC progression through different ways: with the downregulation of the expression of transforming growth factor-β (TGF-β), via the synthesis of pro-inflammatory interleukin (IL) (IL-6 and IL-8) and through the ABC transporters expression, linked to chemoresistance [43] (Figure 1).

Mittica et al. [31] investigated the role of hormone receptors in the development of EOC BMs and observed that patients with AR-negative primary EOC have a 9.5 times greater risk of developing BMs compared to AR-positive EOC. A reduction in the expression of ER was also observed. Interestingly, the hormone receptor expression was also low in metastatic tissue, suggesting a process of neoplastic de-differentiation in this subset of patients. The role of AR as an independent factor was confirmed in a subsequent study by the same group [44]: AR negative EOC had an odds ratio (OR) = 8.33 for brain dissemination, compared to AR-positive EOC; an AR expression <10% significantly correlates with a lower Progression Free Survival (PFS) and a lower brain-specific PFS (see Table 1).

The role of ER was also investigated by Starszewska et al. [32], but no correlation between ER status and the risk of developing BMs in OC or breast cancer was observed. However, in the same study, negative PgR expression was linked to the risk of brain dissemination from breast cancer.

Assessment of hormone receptors in BMs can also be useful to identify possible responders to hormonal therapy. Recently, in BMs from hormone-dependent breast cancer, it has been observed that endocrine therapy correlates with better OS than in those who did not receive the therapy (15 vs 4 months, *p* < 0.001), with no significant difference among patients being treated with AIs, tamoxifen, or fulvestrant [45]. Currently, no cases of BMs from EOC treated with endocrine therapy have been reported; however, there is a rationale for the use of these therapies in EOC treatment. Indeed, phase II and phase III trials on the use of anti-estrogens (tamoxifen and fulvestrant) and aromatase inhibitors (letrozole, anastrozole, and exemestane) in EOC are still ongoing [27,46,47,48]. Interestingly, a study involving 64 patients with recurrent low-grade serous OC treated with hormone therapy (mainly aromatase inhibitor or tamoxifen) showed an objective response rate of 9%; over 60% had a stable disease with a median time to progression of 7.4 months [49].

## 5. BRCA

Both BRCA1 and BRCA2 tumor suppressor genes play an important role in DNA repair via homologous recombination. Cells lacking the homologous recombination pathway are extremely sensitive to DNA damage and are unable to repair DNA double-strand breaks [50,51,52] (see Figure 1). These mutations may result in genomic instability and sustain cancer development. On the other hand, homologous recombination deficiencies can also interfere with cancer cells’ ability to repair DNA-cross links induced by chemotherapy [50,51,52], therefore making them more susceptible to treatment. Germline mutations in BRCA1 and BRCA2 genes account for about 5–10% of all breast cancers and 10–18% of all EOCs [53]. BRCA-deficient women with EOC exhibit increased susceptibility to platinum-based chemotherapy and longer survival [52,54,55,56], although these patients are more prone to developing extra pelvic metastases [57], including BMs. A study on 340 cases of EOCs showed that, out of 7 cases of patients with BMs, 4 had a loss of heterozygosity and 2 had a germline mutation of BRCA1 [58]. The possible role of BRCA1 was also suggested by another study, in which its expression was evaluated in 29 patients with breast cancer and 22 patients with EOC who developed BMs: 65.5% of breast cancer patients and 68.2% of EOC patients showed a BRCA1 protein loss [32]. Ratner et al. [33], analysed a large cohort of EOC patients, which showed a higher prevalence of BMs among patients with the BRCA mutation (14/473, 3%), compared to wild type patients (10/1679, 0.6%), estimating a hazard ratio for developing brain metastasis of 3.84 (95% CI: 1.60–9.22, *p* < 0.001) in mutation carriers (see Table 1). Furthermore, a recent study compared the outcomes of 21 BRCA mutated patients with 42 wild type patients; both groups had BMs and showed better survival in mutation carriers. BMs were more common, but were often isolated and without extracranial involvement [59].

In the landscape of EOC treatment, the introduction of poly ADP ribose polymerase inhibitors (PARPi) resulted in a remarkable improvement in PFS [60,61,62,63,64].

Currently, three PARP inhibitors are approved for the treatment of EOC: olaparib, rucaparib, and niraparib. Olaparib is approved for BRCA mutated patients while niraparib and rucaparib can be used in BRCA WT patients.

Inhibition of PARP leads to the accumulation of DNA damages through different mechanisms: trapping PARP on damaged DNA in order to alter PARP’s catalytic cycle, activating non-homologous end-joining, hindering DNA repair and interfering with BRCA1 recruitment and activity [65,66,67,68,69,70].

Pre-clinical studies have shown how specific PARPi, like niraparib, can cross the blood–brain barrier [71,72] and, in recent years, there have been cases reporting the use of these drugs in this subset of patients: five patients treated with PARPi had BRCA mutation; all of them achieved a progression-free survival of over 10 months [73,74,75,76,77]. Interestingly, a phase II single arm open-label study to evaluate the efficacy of niraparib in patients with BMs is ongoing [NCT04992013].

At present, there are no approved indications for the use of PARPi for the treatment of BMs originating from EOC.

In conclusion, the presence of BRCA mutation is a risk factor for the development of BMs from EOC. Patients may benefit from treatment with PARPi; however, further studies are needed to confirm the therapeutic potential of these drugs.

## 6. MDR1

The multidrug resistance protein 1 (MRP1) is a transmembrane efflux pump member belonging to the ABC (ATP-binding cassette) family of drug transposers encoded by multi drug reactivity 1 (MDR1) gene [78] (see Figure 1). Normally, MDR1 is expressed in various organs, such as intestine, placenta, liver, and brain, where it plays a protective role by reducing the intracellular accumulation of xenobiotic substances [79]. Additionally, MDR1 is involved in the secretion processes of steroid hormones such as glucocorticoids [79]. However, MDR1 overexpression is linked to the development of chemoresistance.

Some cancer cells have inherently high expression of drug efflux transporters, even without previous exposure to cytotoxic drugs. MDR1, in particular, is related to resistance to various chemotherapy drugs, including taxanes, epipodophyllotoxins, vinca alkaloids and anthracyclines [78,80]. MDR1 also plays a pivotal role in the chemoresistance of EOC, not only for the classic taxane-based chemotherapy, but also for PARPi treatment [81,82,83]. In EOC microenvironment, MDR1 overexpression was shown not only in cancer cells, but also in subpopulations of immune cells with pro-inflammatory and pro-tumor activity, such as M2 tumor-associated macrophages, allowing for increased survival of these cells [84]. Given these premises, it is clear how high MDR1 expression is significantly associated with poor outcomes in patients with EOC [85] and could be considered a useful biomarker for prognosis and response to chemotherapy.

MDR1 expression has also been investigated as a possible promoter of BMs in EOC. Matsuo et al. [34] investigated a series of 309 cases selected for epithelial ovarian, fallopian tube, and primary peritoneal cancers, 5 of which developed BMs. The expression of MDR1 in primary cancer from patients who developed brain metastasis was observed in 80% of the cases, and this proportion was significantly higher than cases that developed relapses in other locations, such as abdomen or pelvis (24.3%), liver or spleen (18.4%), and lung (4.2%) (*p* = 0.004, Table 1). The expression of MDR1 in the primary tumor suggests greater resistance to adjuvant chemotherapy and implies a greater risk of developing distant metastases. Unfortunately, the expression of MDR1 in brain metastasis has not been evaluated; however, preclinical models of brain metastasis originating from breast cancer suggest the presence of drug efflux transporters in the blood–tumor barrier and in adjacent tumour cells [86]. Therefore, MDR1 could represent a predictive biomarker for the development of BMs from EOC, but further research is needed in order to better understand its role in the development of brain recurrence and in the biology of metastatic tumours.

## 7. PD-1/PD-L1

The PD1/PD-L1 pathway plays a critical role in limiting activation of immune system [87].

Programmed cell death 1 (PD-1) interacts with its ligands PD-L1 [88] and PD-L2 [89], inhibiting T cell receptor (TCR) signalling, leading to reduced T cell proliferation and cytokine production and increasing susceptibility to apoptosis (Figure 1). The PD-L1/PD-1 interaction block using antibodies against either molecule can reconstitute T cell function, leading to an amplification of anti-tumor immune responses. [87,90]

Restoring the immune system against different types of cancers has been proven to be a promising strategy in recent years [91,92].

However, regarding gynaecological tumors, the role of immunotherapy has so far been limited [93,94,95,96]. The efficacy of immunotherapy has been tested in ovarian cancer; however, the results were not as expected: the main phase III immunotherapy trials conducted in ovarian cancer yielded negative results [97,98,99]. Currently, there are no approved immune therapies [100] and PD-L1 has not been shown to be an effective biomarker in predicting response to immune checkpoint inhibitors (ICIs) [98,100,101,102,103,104].

Little is known about PD-L1 expression and microenvironment in brain metastases from EOC. Choi et al. investigated immunologic and genomic profiles of two cases of primary ovarian tumors and their matched brain metastases: PD-L1 expression in the brain tissue was higher than that in the primary tumors, while the overall density of immune infiltrates was not significantly different (Table 1) [35]. A paper from Gill et al. showed slightly different results, observing that an active tumor immune microenvironment is present with similar distribution in the primary disease site and BMs from patients with gynecologic malignancies (of which, 23 were ovarian primaries, 52.3%) [105].

These data could encourage the use of checkpoint inhibitors in BMs from EOC but evidence is still too limited.

## 8. Other Biomarkers

Several studies have tried to identify new biomarkers that can predict the development of BMs from EOCs. Yoshida et al. [106] analysed the protein expression of three cases of BMs from gynaecological cancers, through a proteomic analysis, identifying 129 proteins. The authors found that in endometrial and EOC BMs, the expression of alpha-enolase (ENO1) and triosephosphate isomerase (TPI-1) was higher and the expression of Transgelin-2 (TAGLN2) was lower compared to primary cancers, suggesting a role in development and progression of BMs. Another biomarker that has been related to the development of brain metastases is DNA S-phase fraction. The study conducted by Matsuo et al. [34] observed that the level of DNA S-phase fraction is higher in BMs (14.9%) than in other metastatic sites (liver and/or spleen 8.2%, lung 11.4%). Human epidermal growth factor receptor 2 (HER2) is a marker whose overexpression is linked to worse survival in EOC; however, reduced or absent expression was observed in primary tumors developing BMs [31].

Table 2 contains the main studies that have evaluated the predictive role of biomarkers of brain diffusion from ovarian cancer.

## 9. Conclusions

The development of BMs from EOC is a rare event; however, it should not be underestimated given the growing incidence and difficulty in management. This review describes the current biomarkers landscape of patients with EOC who develop BMs. The identification of a biopredictor of BMs development from EOC may help personalizing follow-up programs, for examples by adding a head computed tomography scan (CT-scan), usually not included in follow-up, according to the risk of CNS evolution. There is growing evidence on the role of some biomarkers, such as the presence of BRCA mutation, that can help identify populations of patients at risk of this complication, while others, such as MDR-1 and hormone receptors, can also be potential therapeutic targets. Unfortunately, all data is derived from retrospective studies, often with limited case numbers; we need prospective multicentre studies to clarify the role of these markers.

## Figures and Tables

**Table 1:** Principle biomarkers of brain spread from EOC.

	Expressed on OC/BM	Methods	Sample Size (n)	Predictive Role of Brain Spread	Ratio for Developing Brain Metastasis	References
ER	Both	IHC	40	No		[31]
	EOC	IHC	22	No		[32]
PgR	Both	IHC	40	No		[31]
AR	Both	IHC	40	Yes (the absence of AR on EOC)	OR 8,33 (95% CI: 2.15–32.29)	[31]
HER2	Both	IHC	40	No		[31]
BRCA	EOC	Somatic or germline mutation	4233	Yes (the presence of BRCA mutation)	HR 3.84	[33]
					(95% CI: 1.60–9.22, *p* < 0.001)	
MDR1	EOC	IHC	309	Yes (the presence)	NE	[34]
PD-L1	Both	IF	2	No (expression in BMs was higher than EOC)		[35]

ER estrogen receptor; EOC epithelial ovarian cancer; PgR progesterone receptor; AR androgen receptor; HER2 Human Epidermal Growth Factor Receptor 2; BRCA breast cancer gene; MDR multi drug reactivity 1; PD-L1 Programmed cell death ligand 1; IHC immunohistochemistry; IF immunofluorescence; BMs brain metastases.

**Table 2:** Main studies that have evaluated the predictive role of biomarkers of BMs from ovarian cancer.

	Biomarkers	Methods	Sample Size (n)	Conclusions	References
Mittica et al.	ER PgR AR HER2	IHC	40	The absence of AR on EOC is predictive of BMs	[31,44]
Szarszewska et al.	ER	IHC	22	No predictive role of BMs development	[32]
Ratner et al.	BRCA	Somatic or germline mutation	4233	The presence of BRCA mutation is predictive of BMs	[33]
Matsuo et al.	MDR1	IHC	309	The presence of MDR1 is predictive of BMs	[34]
Choi et al.	PD-L1	IF	2	Expression of PD-L1 in BMs was higher than EOC	[35]

ER estrogen receptor; EOC epithelial ovarian cancer; PgR progesterone receptor; AR androgen receptor; HER2 human epidermal growth factor receptor 2; BRCA breast cancer gene; MDR multi drug reactivity 1; PD-L1 programmed cell death ligand 1; IHC immunohistochemistry; IF immunofluorescence; BMs brain metastases
